# Therapeutic Applications of Nanoformulated Resveratrol and Quercetin Phytochemicals in Colorectal Cancer—An Updated Review

**DOI:** 10.3390/pharmaceutics16060761

**Published:** 2024-06-04

**Authors:** Dhanalekshmi Unnikrishnan Meenakshi, Gurpreet Kaur Narde, Alka Ahuja, Khalid Al Balushi, Arul Prakash Francis, Shah Alam Khan

**Affiliations:** 1 College of Pharmacy, National University of Science and Technology, Muscat PC 130, Oman; gurpreetdrd@rediffmail.com (G.K.N.); kbalushi@nu.edu.om (K.A.B.); shahalam@nu.edu.om (S.A.K.); 2 Centre of Molecular Medicine and Diagnostics (COMMAND), Saveetha Dental College and Hospitals, Saveetha Institute of Medical & Technical Sciences, Saveetha University, Chennai 600077, India; fdapharma@gmail.com

**Keywords:** polyphenols, resveratrol, quercetin, colon cancer, MAPK, NF-κB, AKT, nanoformulations

## Abstract

Natural compounds such as polyphenols play several positive roles in maintaining the oxidative and inflammatory capacity of cells, which leads to their potential use as anticancer therapeutics. There is promising evidence for the in vitro and in vivo anticancer activity of many polyphenols, including resveratrol and quercetin, specifically in the treatment of colorectal cancer (CRC). There is a clear association between resveratrol and quercetin in interfering with the mechanistic pathways involved in CRC, such as Wnt, P13K/AKT, caspase-3, MAPK, NF-κB, etc. These molecular pathways establish the role of resveratrol and quercetin in controlling cancer cell growth, inducing apoptosis, and inhibiting metastasis. The major bottleneck in the progression of the use of resveratrol and quercetin as anticancer therapeutics is their reduced bioavailability in vivo because of their rapid metabolism in humans. Recent advancements in various nanotechnological formulations are promising for overcoming these bioavailability issues. Various nanoformulations of resveratrol and quercetin have shown an optimistic impact on reducing the solubility and improving the stability of resveratrol and quercetin in vivo. A combinatorial approach using nanoformulations of resveratrol with quercetin could potentially increase the impact of resveratrol in controlling CRC cell proliferation. This review discusses the mechanism of resveratrol and quercetin, the two bioactive polyphenolics, in colon cancer, with an emphasis on various types of nanoformulations of the two molecules targeting colon cancer. It also explores the synergistic effect of combining resveratrol and quercetin in various nanoformulations, targeting colon cancer. This research delves into the enhanced pharmacokinetics and potential chemotherapeutic benefits of these bioactive polyphenolics when used together in innovative ways.

## 1. Introduction

Colorectal cancer (CRC) is the most common cancer diagnosed and the second deadliest malignancy, irrespective of the gender. According to the world health organization (WHO), more than 1.9 million new cases and more than 930,000 deaths due to CRC were estimated to have occurred worldwide in 2020 [1]. CRC is a multifactorial disease, exhibiting both strong environmental associations and genetic risk factors. The epithelial cells of the colorectal mucosa can undergo hyperplasia, atypical hyperplasia, and adenomas, that can eventually develop into carcinoma [2]. Several cellular signaling pathways that regulate cell proliferation, differentiation, apoptosis, and survival are involved in CRC onset, such as epidermal growth factor receptor (EGFR)/mitogen-activated protein kinase (MAPK), wingless-related integration site (Wnt)/β-catenin, phosphoinositide 3-kinase (PI3K), transforming growth factor-β (TGF)-β, neurogenic locus notch homolog protein (Notch), and nuclear factor (NF)-κB [3]. Currently, treatments for CRC include surgical resection, chemotherapy, targeted therapy, immunotherapy, gene therapy, and combination therapies. For decades, commonly used CRC chemotherapeutic agents included 5-Fluorouracil (5-FU), Irinotecan, Oxaliplatin, Calcium folinate, Capecitabine, S-1 (Tegafur/gimeracil/oteracil), and TAS-102 (Trifluridine/Tipiracil) [3]. Chemotherapy although effective, is associated with many side effects, and recent advancements such as monoclonal antibody or immunotherapy is not generally affordable for most people because of financial constraints [4]. Hence, chemotherapeutics providing the best possible outcomes, lower toxicity, and improved quality of life (QoL) are needed. The void in regard to the availability of treatments with fewer side effects for such deadly diseases can be filled by phytochemicals, which have shown enormous therapeutic potential in experimental studies. 

Over the past several years, the potential of a wide variety of multi-targeted phytochemicals such as curcumin, genistein, berberine, resveratrol, quercetin, boswellic acid, epigallocatechin gallate (EGCG), garcinol, piperine, tocotrienol, honokiol, capsaicin, betulinic acid, apigenin, withaferin, diosgenin, etc. have been extensively investigated [4,5]. Among these bioactive natural compounds, polyphenols are gaining increased popularity because of their low side effects, bioavailability traits, and promissory beneficial effects, including antioxidant, antiinflammatory, antiaging, and anticancer properties [5]. Quercetin and resveratrol are two biologically active polyphenolic phytochemicals that have been demonstrated to be effective in the prevention and treatment of cancer. The unique properties of resveratrol and quercetin, such as their antioxidant and antiinflammatory properties, make them promising candidates for cancer prevention and treatment [6,7]. However, their limited bioavailability poses a significant challenge to the scientific community [6,7]. Building upon the scientific interest in resveratrol and quercetin for their chemotherapeutic effects against specific types of cancers, researchers are increasingly focusing on novel drug delivery formulations to overcome their poor solubility and bioavailability. These repurposed novel formulations may maximize the therapeutic efficacy of resveratrol and quercetin by enabling tailored delivery to cancer cells, in addition to improving the stability and absorption of these nutrients. This research highlights the significance of formulation strategies to effectively utilize the therapeutic potential of quercetin and resveratrol in the fight against cancer.

**Figure 1:**
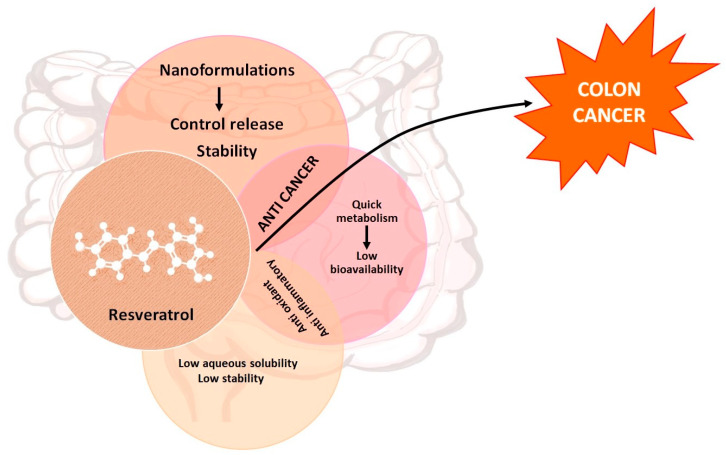
Resveratrol exhibits limitations for its therapeutic use in regard to stability, solubility, and bioavailability. Encapsulating resveratrol in nanoformulations is a strategy to utilize its antioxidant, antiinflammatory, and anticancer properties more effectively. Resveratrol nanoformulations can be promising chemotherapeutics in the treatment of CRC.

Resveratrol (3,5,4′-trihydroxy-*trans*-stilbene) is a natural polyphenol compound found abundantly in fruits such as apples, grapes, pears, plums, and cherries; in vegetables such as spinach, broccoli, chicory, flax, and onion; and in some condiments and beverages, including tea and red wine [8]. As shown in Figure 1, resveratrol is a weak acid consisting of two phenols joined together by an unsaturated alkene bridge. Due to deprotonation of hydroxyl groups present in the compound, resveratrol has three acidic dissociation constants (pK_a1_, pK_a2_, pK_a3_ = 8.8, 9.8 and 11.4). This natural compound exhibits poor water solubility (0.05 mg/mL), with a partition coefficient of 3.1. The presence of double bonds allows for two geometrical isomers of resveratrol (i.e., *cis*- and *trans*-resveratrol). In the plant, it can exist in the free form or in the form of glycoside [9]. Resveratrol has been of interest for more than eight decades because of its potent anti-cancer properties [6].

There is a vast amount of literature underlining the mechanism by which the efficacy of resveratrol is proven beyond a doubt [6,7,8,9]. The plasma concentration of resveratrol is much lower than the inhibitory dose required for its therapeutic activity. The low C_max_ of resveratrol may be mainly due to its rapid metabolism rather than to its poor water solubility. Also, as reported by Kossi et al., resveratrol has been shown to bind extensively and tightly to human serum albumin, further limiting its bioavailability to target tissue [10]. Out of the many possibilities, i.e., altering the chemical structure of resveratrol or using its derivatives, a feasible option is to employ the nanoformulation of resveratrol. Howells et al. reported encouraging results using increased plasma levels of resveratrol by employing a micronized formulation [11]. Studies have also shown that the chemo-preventive and chemo-therapeutic action of resveratrol can be improved by nanoformulations [12].

**Figure 2:**
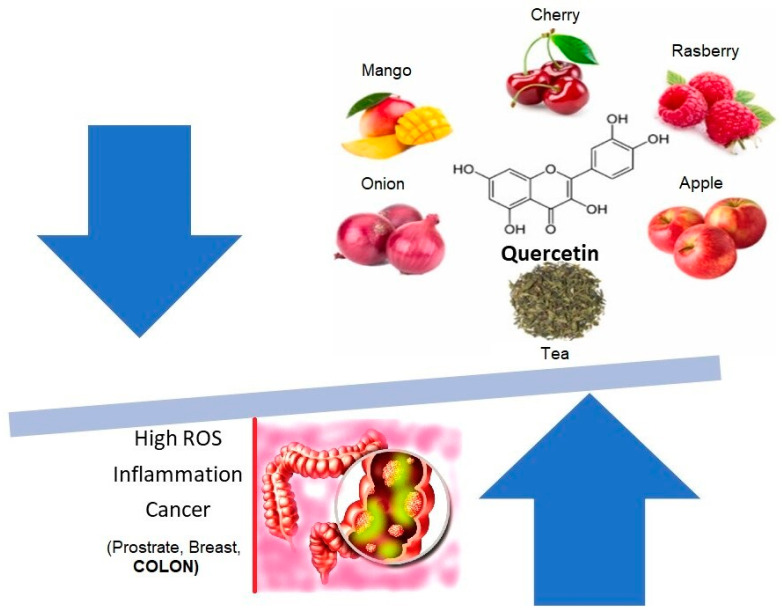
Quercetin, a flavanol found in many fruits, is a powerful radical scavenger and is a potential candidate for chemotherapy.

Quercetin, a flavanol, is another interesting bioactive polyphenolic compound possessing a broad spectrum of biological activities. It has a unique structure, with five hydroxyl groups responsible for biological activity (Figure 2). Strong intramolecular H-bonding is exhibited by quercetin, which helps to explain the biological multi-functionality of the compound and enables it to form complexes with metals [12].

The powerful antioxidant properties of quercetin have been attributed to the hydroxyl groups in the A ring and a catechol group in the B ring [13]. Because of this ability, quercetin can decrease the activities of enzymes by removing free oxygen species that are already present in the body through the transfer of hydrogen or electrons or by the chelation of metal ions. This process of scavenging reactive oxygen species (ROS) helps to minimize inflammation and protects cells from the oxidative stress. Quercetin is found abundantly in various vegetables and fruits, such as berries, lovage, capers, cilantro, dill, apples, and onions [14]. The solubility of quercetin is about 0.09 μg/mL in water, with a limited oral bioavailability [15]. Although it can easily cross the intestinal cells, it cannot pass through the mucous layer. The absorbed amount of quercetin in healthy individuals has been found to be very low, between 3–17% [16], while in vivo studies showed maximum metabolism of the molecule—almost 98%—within one hour of administration [17]. Quercetin occurs in conjugated (glycated) and unconjugated forms. In 2014, Alrawaiq and Abdullah reported that the plasma concentration of the unconjugated form, which is absorbed through the intestine, is quite small [18]. Graefe et al. also studied the pharmacokinetic properties of quercetin and found that the plasma concentrations were only about 2.3 μg/mL in 48 min [19]. In a limited human trial, quercetin, along with curcumin, yielded encouraging results in reducing the size and number of adenomatous polyps [20]. Numerous in vivo studies have shown the impact of quercetin in providing positive outcomes in colon cancer [7].

Over the past three decades, pharmaceutical scientists have shown a great deal of interest in investigating the potential of natural substances like quercetin and resveratrol in the prevention and treatment of cancer. Besides being nontoxic to normal cells, these natural bioactive compounds could suppress or delay the progression of cancer. Therefore, developing novel drug delivery strategies to increase the effectiveness and bioavailability of these chemicals has been a major focus of pharmaceutical development research. Innovative formulations combining resveratrol and quercetin show considerable potential for synergistic effects that may enhance each compound’s individual therapeutic benefits. Through the investigation of novel drug delivery methods, scientists hope to enhance the solubility, stability, and targeted distribution of bioactive components. This strategy not only improves the pharmacokinetics of quercetin and resveratrol but also creates new avenues for their use in the prevention and treatment of cancer, as discussed throughout this article. Based on the above-mentioned rationale, the present review discusses the mechanism of resveratrol and quercetin, the two bioactive polyphenolics, in colon cancer, with an emphasis on various types of nanoformulations of the two molecules targeting colon cancer. The novelty in this review work lies in the exploration of the synergistic effect of combining resveratrol and quercetin in various nanoformulations targeting colon cancer. This research delves into the enhanced pharmacokinetics and potential chemotherapeutic benefits of these bioactive polyphenolics when used together in innovative ways. When considering the trend in novel formulation, these compounds could possibly be encapsulated or incorporated to improve their absorption and distribution to the target cells, thus enhancing their efficacy in cancer prevention and treatment.

## 2. Resveratrol and Quercetin: Mechanism of Action in Colon Cancer

**Figure 3:**
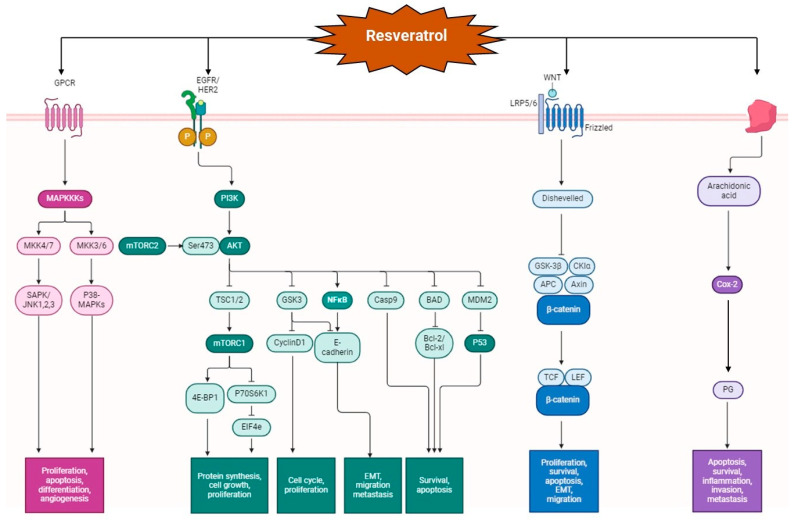
Various molecular mechanisms that are altered by resveratrol. Resveratrol acts as an AKT inhibitor and in turn, the NF-κB pathway, resulting in the inhibition of metastasis, cell proliferation, and cell growth. The AKT pathway is also responsible for cell survival via the *p53* gene; resveratrol inhibits cell growth and increases apoptosis. The Wnt-β catenin pathway is disturbed by degradation of β-catenin in the presence of resveratrol, thus inhibiting cell proliferation and migration. Resveratrol also inhibits the COX-2 pathway, resulting in decreased inflammation and neoplastic growth. The MAP kinases responsible for differentiation and angiogenesis are inhibited by resveratrol.

Resveratrol and Quercetin exert its effect by virtue of its antiinflammatory, antioxidant, and apoptotic properties. CRC is marked by chronic inflammation. Recent studies have shown that exposure of intestinal cells to cytokines can mobilize inflammatory pathways,, such as MAPKs (mitogen-activated protein kinase), JAK-STAT (Janus kinase/signal transducer and activators of transcription) and NF-κB (nuclear factor kappa-light-chain-enhancer of activated B cells) cascades, raise the expression of proinflammatory enzymes, and induce the generation of proinflammatory mediators and the production of ROS [21]. As shown in Figure 3, resveratrol can exert an antiinflammatory response by decreasing proinflammatory mediators, such as TNF-α (tumor necrosis factor alpha) and IL-1β (interleukin-1 beta); proinflammatory enzymes, such as iNOS (inducible nitric oxide synthase) and COX-2 (cyclooxygenase-2); and inflammatory signaling pathways, such as NF-κB [22]. Another study showed that resveratrol inhibited TNF-β and increased apoptotic factors such as cleaved caspase-3. It also showed that resveratrol decreased NF-κB activation and the gene products dependent on it, such as MMP-9 (matrix metalloproteinase) and CXCR4 (chemokine receptor type 4) [23]. There is empirical evidence that resveratrol can inhibit MMP-9 and VEGF, preventing metastasis and angiogenesis [24]. Resveratrol can inhibit endothelial cell adhesion and migration by reducing MMP-2 activity during neo-angiogenesis in both in vivo and ex vivo assays [25]. Resveratrol at concentrations of 12.5, 25, and 50 μM inhibited the migration of MDA231 cells via the EMT pathway (epithelial–mesenchymal transition). The EMT pathway has often been associated with tumor invasion and metastasis in ovarian, breast, colon, lung, prostate, oral, and liver cancer, etc. [26].

Other antiangiogenic activities exhibited by resveratrol include the inhibition of hypoxia-inducible factor-1alpha (HIF-1alpha) accumulation and an increased expression of thrombospondin-1 (TSP1), a natural inhibitor of angiogenesis [27]. In human and animal models of CRC, a significant role of nitric oxide (NO) in colon tumorigenesis has been indicated due to the increased activation and expression of iNOS. Several studies have also reported that resveratrol decreased iNOS expression in colon cancer cells. However, its primary mechanism is still unclear [28]. Generally, NO and PG (prostaglandins) play an essential role in cell proliferation and angiogenesis, triggering tumor growth and metastasis. Resveratrol has been shown to decrease the expression of PGs by inhibiting a COX-2 enzyme, which catalyzes the arachidonic acid conversion into PGs [27].

The potential use of resveratrol in cancer treatment could also be justified by its capacity to cause autophagic cell death by inducing overstimulation of autophagy in cells that are faulty regarding apoptosis. The induction of autophagy through increased sirtuin1 (SIRT1) activation and expression, the inhibition of the protein kinase B/mammalian target of rapamycin (AKT/mTOR), and the activation of p38-MAPK are the most well-known signaling pathways for resveratrol [29]. Furthermore, it has been demonstrated that resveratrol’s anti-tumor action at 200 μM is enhanced by SIRT1 inhibitors (nicotinamide: 5 mmol/L) and autophagy inhibitors (3 methyl adenine: 10 mmol/L). By binding with αvβ3 integrin and activating ERK 1/2 (extracellular signal-regulated kinase) via MAPK-kinase, resveratrol has been shown to be an effective apoptosis inducer in cellular regulative mechanisms [30]. MAPK-kinase is a crucial protein regarding the interaction of cancer cells. However, this has only been documented to occur for brief activation and at concentrations of 1 pM−10 μM. As shown in Figure 3, *Akt,* a proto-oncogene that promotes cell cycle progression and inhibits apoptosis, is highly activated in 60–70% of human CRC [31]. Targeting AKT signaling is a promising option for locating novel molecular targets for cancer therapy. MK-2206, an AKT kinase inhibitor, has shown promising preclinical anticancer activity [32,33], entering phase II clinical trials for metastatic breast cancer and CRC in 2017. The transcription factor that carries the signal of the AKT pathway is STAT 3 (signal transducer and activator of transcription). It regulates the expression of essential pro-invasive factors, such as matrix metallopeptidases, HSP 70 (heat shock protein), and HSP90 [34]. A study by Li et al. demonstrated that resveratrol could inhibit cell proliferation and promote cell apoptosis via the STAT3 signaling pathway, where AKT served as an upstream regulator of STAT3 in CRC [35]. Resveratrol, in a dose-dependent manner, results in the suppression of the Wnt (wingless-related integration site) signaling pathway, which is one of the critical pathways in several severe diseases like cancer. Resveratrol decreases the expression of the *Wnt* target genes, including cyclin D1, and conducts and suppresses the development of Wntinduced cells and Wnt-driven CRC cells [36]. Additionally, the administration of resveratrol has been shown to regulate the enzymatic activity of PDH (pyruvate dehydrogenase) in colon cancer [37]. The activity of the PDH complex was measured in colon cancer cells following treatment with 10 μM resveratrol for 48 h using [^14^C]-pyruvate, and it was observed that the activity of the PDH complex was enhanced by 2.6-fold. Likewise, a recent study revealed similar trends in colon cancer cell proliferation following the use of resveratrol. It was demonstrated that at concentrations of 1–10 μM, resveratrol enhanced cell proliferation after 96 h of treatment, whereas at 50 and 100 μM, it decreased the cell number, suggesting cytotoxicity [38].

Quercetin has many effects on CRC that are very similar to those of resveratrol. Among the anti-carcinogenic activities of quercetin, the most remarkable effects described for CRC are the inhibition of cellular proliferation and growth, cell cycle arrest, the induction of apoptosis, a reduction in tumor size, a decrease in number of tumor nodules, the suppression of metastasis, a decrease in inflammation, a decrease in ROS, and a reduction in multidrug resistance. In addition, quercetin exhibits many other beneficial properties that make it an effective supplement to the daily diet, including antiinflammatory, antihypertensive, and antithrombotic effects; antiatherosclerosis properties; and antiarrhythmic activity. Therefore, quercetin has attracted attention from researchers as an adjuvant agent due to its antitumor, antioxidant, cytoprotective, and antiproliferative properties [39]. One of the most essential features of quercetin is its pro-apoptotic effect, which is caused by increasing pro-apoptotic molecules such as P53, BAX, caspase-3, and caspase-9 or by stimulating the mitochondrial apoptosis pathway or decreasing antiapoptotic proteins [40,41,42,43]. Many research works have confirmed that quercetin can stabilize P53 levels and increase phosphorylation [44,45,46,47]. In malignant cells, *p53* genes can be blocked or mutated, causing the loss of functions [48]. 

**Figure 4:**
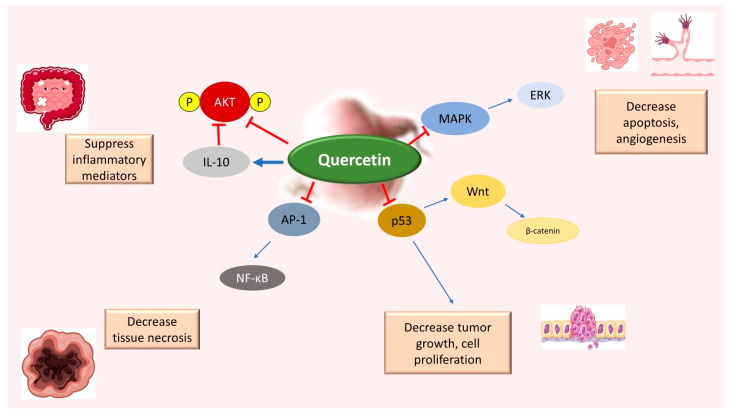
Quercetin inhibits various important pathways (AKT, NF-κB, P53, and MAPK) involved in the progression of colon cancer (blue arrows indicate regular pathways). As shown by in vitro studies in different colon cell lines, quercetin can (denoted by red inhibitory arrows) suppress inflammation, reduce tumor growth, and cell proliferation, and decrease apoptosis and angiogenesis.

Another antioxidant function of quercetin is to quench ROS, thereby preventing ROS-mediated DNA damage [49,50]. Therefore, querectin has the potential to be used in cancer treatment because of its ability to regulate the cell cycle, its antioxidant effects, P53 stabilization, and apoptosis induction. As shown in Figure 4, quercetin is mainly involved in influencing the Wnt/β-catenin, PI3K/AKT/mTOR (phosphoinositide 3-kinase/protein kinase B/ mammalian target of rapamycin), MAPK/ERK (extracellular signal-regulated kinase), MAPK/JNK (c Jun N terminal kinase), MAPK/P38, P53, and NF-κB pathways. Generally, β-catenin levels are maintained at low levels in the cells; however, Wnt ligand signaling results in the accumulation of β-catenin in the cytosol, which after translocation to the nucleus, can trigger the oncogenic mechanism [51,52]. Quercetin has shown a downregulation of the Wnt/β-catenin pathway in the HT-29 cell line. Deregulating the PI3K/AKT pathway is a critical event in cancer pathogenesis [53,54]. The PI3K enzyme catalyzes the conversion of phosphatidylinositol (3,4)-bisphosphate into phosphatidylinositol (3,4,5)-trisphosphate, therefore triggering the phosphorylation of AKT, followed by translational discrepancies [55]. Quercetin inhibits the activity of the AKT enzyme, as demonstrated in several CRC representative cell lines, such as HT-29, Caco-2, DLD-1, and HCT-15. The MAPK pathway is an indirect target of quercetin, and it has been shown that the concentration of p-STAT3 decreases upon media supplementation with the flavonol for the Caco-2 and DLD-1 cell cultures [56]. Quercetin can induce apoptosis in CRC cells. As shown in one study, in the human CRC cell lines obtained from patients with microsatellite instability (MSI), quercetin stimulated 5-fluorouracil-induced apoptosis in a P53-dependent way [52]. Common chemical properties of resveratrol and quercetin that contribute towards the pharmacological mechanisms targeting colon cancer are consolidated and depicted in Table 1.

The overall anticancer efficacy of resveratrol and quercetin is very low, even though they have distinct molecular anticancer pathways. The bioavailability of these phytoconstituents may be a contributing factor, as they may not be effectively absorbed and dispersed throughout the body to achieve their intended effects. Furthermore, a combination of many chemicals, bioenhancers, or targeted therapies may be necessary to effectively combat the disease due to the complexity of cancer and the multiplicity of pathways involved in its evolution. It is also possible that inter-individual variation in disease environment and response could impact the overall efficacy. Additional investigation, formulation approaches, and clinical trials are required to completely understand and optimize the efficacy of resveratrol and quercetin against CRC.

## 3. Challenges Regarding Oral Administration of Resveratrol and Quercetin

The numerous studies conducted in vitro and in vivo are a testament that resveratrol and quercetin are promising candidates for the treatment of CRC. Despite the high curative potential, their low solubility and poor pharmacokinetic properties hinder the proper gastrointestinal (GI) absorption of these phytonutrients, leading to poor bioavailability. They are hydrophobic in nature. Resveratrol is highly soluble in alcohol and PEG-400 [57]. The bioavailability of resveratrol is also limited because of its extensive metabolism in the intestine and liver [59]. As reported by Patel et al., it was shown that resveratrol can accumulate in the GI tract, probably because of its accumulation in the epithelial cells [60]. Although resveratrol is well absorbed, it has low bioavailability, resulting in low plasma concentrations, giving rise to limited systemic distribution and concentrations that are not high enough at specific active sites to produce significant pharmacological effects. The bioavailability of resveratrol, which is less than 1%, is an issue, as it has been shown that resveratrol has a C_max_ of 2.4 μM, and the T_max_ is 1.5 h, which is much lower than the inhibitory concentrations (IC_50_) required for most of the cancer cells in in vitro systems [61]. On the other hand, in human colon cancer trials, a micronized formulation of resveratrol was found to have a C_max_ of 8.51 μM, which is an encouraging value in regard to the chemotherapeutic potential of resveratrol [11]. Similarly, quercetin is sparingly soluble in water, but its solubility increases marginally by increasing the water temperature. Quercetin is soluble in acetone, alcohol, and other organic solvents [58,62]. The bioavailability of flavonoids is subject to the form in which they are administered. Human studies deciphering the bioavailability of quercetin and kaempferol suggest that some conjugated forms exhibit higher bioavailability than the free forms in plants [63]. It has been reported that the absorbed quercetin in healthy volunteers was between 3% and 17% after administration of 100 mg/kg of quercetin. After a single oral treatment of rats with 10 mg quercetin/200 g body weight, 93% of quercetin was metabolized an hour later. The two forms of quercetin—conjugated (glycoside) and unconjugated—have a plasma concentration of 3.5–5.0 μmol/L and <0.33 μmol/L, respectively. The challenging aspect is that the form of quercetin absorbed by the intestine is the conjugated form [64]. The pharmacokinetic properties of quercetin studied by maintaining a dose level of up to 200 mg demonstrated the C_max_ and T_max_ of quercetin to be 2.3 ± 1.5 µg/mL and 0.7 ± 0.3 h, respectively [65].

**Figure 5:**
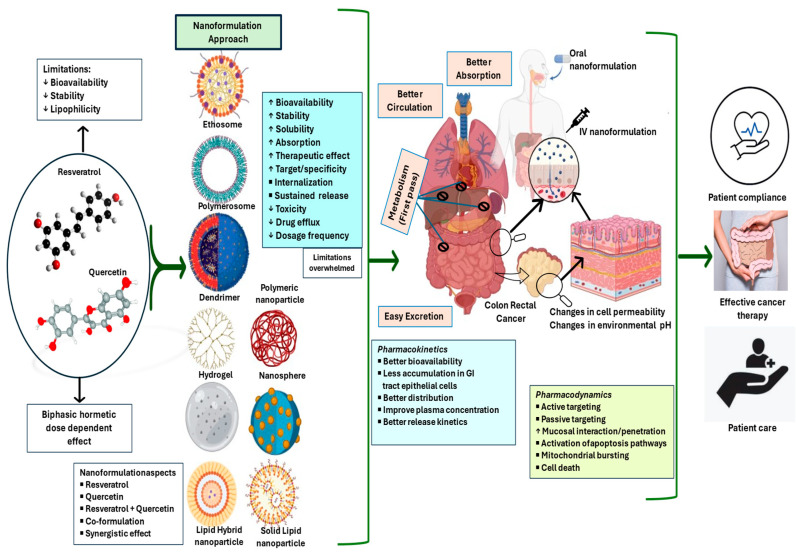
Nanoformulation approach to overcome the limitations of phytoconstituents. This figure illustrates the pharmacokinetic and pharmcodynamic limitations associated with resveratrol and quercetin and the strategies for overcoming these limitations using a nanoformulation approach. On the left side of the figure, the limitations of resveratrol and quercetin are depicted. These factors contribute to low bioavailability and suboptimal therapeutic outcomes. On the right side of the figure, the concept of nanoformulations is introduced as a solution to overcome these limitations. The figure further illustrates how nanoformulations protect the entities from degradation and improve their absorption and distribution in the body. By utilizing nanoformulations, the drug can bypass biological barriers, achieve sustained release, and accumulate at the desired site of action, thereby enhancing its efficacy and reducing the required dosage and frequency of administration. Overall, the figure highlights the transformative potential of nanoformulations in overcoming the limitations of resveratrol and quercetin and improving the pharmacokinetic and pharmacodynamic parameters for effective treatment and better patient outcomes.

The adversity of bioavailability can be addressed by formulating the phytoconstituents as nanoparticles. It has been shown that reducing the size to nanometres improves the solubility and enhances the dissolution and bioavailability of phytoconstituents. It also protects from toxicity and targets the site where the drug is to be delivered [66]. As shown in Figure 5, the nanoformulation approach helps to overcome the pharmacokinetic and pharmacodynamic limitations of phytoconstituents. While formulating phytoconstituents, there are many key points to be considered, the foremost being the stability of the active ingredient. There are other factors to be considered, such as the cost of preparation, biocompatibility, the target site and disease, entrapment efficiency, increased retention time, release kinetics, etc.

## 4. Chemotherapeutic Applications of Various Nanoformulations of Resveratrol and Quercetin in CRC Therapy

**Figure 6:**
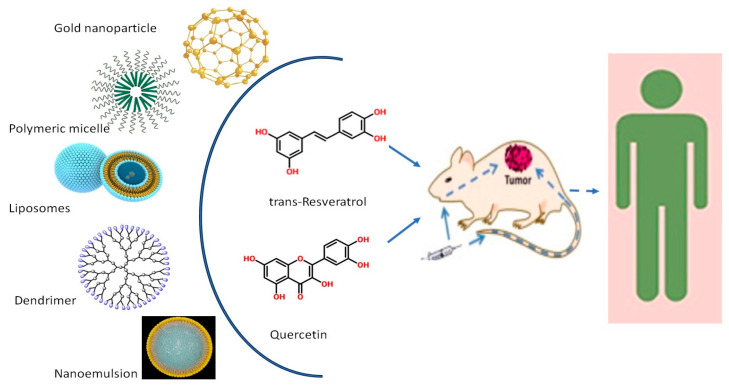
Different nanoformulations used to encapsulate resveratrol and quercetin in targeting colon cancer in vivo in animal models. Based on the established anti-cancer therapeutic impact, the in vivo studies can be extended to human clinical trials.

There is a plethora of literature available on the formulation of resveratrol and quercetin. The major formulations of resveratrol and quercetin reported for CRC therapy include liposomes, nanoemulsions, nanoparticles, and dendrimers. A detailed mapping of various nanoformulations and their applications is illustrated in Figure 6.

### 4.1. Liposomes

Liposomes are biocompatible carriers that can be prepared from lipids with tunable physicochemical properties and loaded with compounds of different lipophilic-hydrophilic natures [67]. Liposomes also enable the slow release of the drug at the target site over prolonged periods of time. Moreover, it has been shown that its association with liposomes is an effective way to protect resveratrol from light and other degradative processes [68]. The liposomal delivery system not only improved the solubility issue for resveratrol, but also increased its anti-tumor effect [69]. Liposomal carriers are also effective in reducing the therapeutic dosage, which in turn, keeps the side effects of the drug in check [70]. Quercetin has been formulated as a liposomal nanocarrier by various research groups. The physical properties of quercetin liposomes depend greatly on the kind of lipids used [71]. Additionally, it has been shown that coating the liposome with polyethylene glycol (PEG) increases the tumor targeting effect [72].

Recently, it was empirically shown that resveratrol-loaded liposomes can effectively destroy the tumor microenvironment in colorectal cancer in vitro. Dana et al., 2023, generated cancer-associated fibroblasts (CAF) containing tumors using a monoculture of HT-29 tumor spheroid and a co-culture of HT-29 and MRC-5 tumor spheroid. Further treatment with resveratrol liposomes at a 50–100 µM concentration was found to specifically inhibit the spheroid growth of CAF containing tumors. This study paves the way for the promising therapeutic use of resveratrol liposomes in the future [73].

Quercetin liposomes prepared by thin film hydration were evaluated for apoptosis in SW48 colon cancer cell lines. The study findings revealed that the liposomes had a doubled apoptotic effect relative to that of free quercetin. The cytotoxicity effect, along with a decrease in EGFR expression, was also found, thereby establishing a promising role of quercetin nanoliposomes in the treatment of colon cancer [74]. Melchior et al. also demonstrated that quercetin-loaded liposomes prepared by high-pressure homogenization showed excellent stability and sensitivity, as well as a significant cytotoxic effect on the HCT-116 *p53*^+/+^ cell line, a wild-type cell line for P53 expression. The results showed that the HCT-116 *p53*^+/+^ cell line was significantly more sensitive to quercetin-loaded liposomes than to free quercetin. A dosage greater than or equal to 50 μM of quercetin liposomes was found to impact the cell metabolism by at least 2-fold [75].

### 4.2. Polymeric Micelles

Polymeric micelles are a promising approach in which amphiphilic block copolymers composed of hydrophilic and hydrophobic segments can self-assemble into polymeric micelles at a concentration above their critical micelle concentration (CMC). They spontaneously self-assemble into nano-sized constructs [76]. Sudha et al. have shown the effect of resveratrol-loaded nanoparticles formulated in polymeric micelles which could reduce the tumor size in an orthotopic tumor transplant in mice. The results clearly indicated that the PLGA resveratrol nanoparticles exhibited greater efficacy in reducing the tumor size compared to that of resveratrol alone. An approximate 20% reduction in tumor mass was found in mice treated with resveratrol polymeric micelles. The study recommends the use of resveratrol nanoparticles as a therapeutic control in colon cancer, since the bioavailability increases almost two-fold for resveratrol micelles relative to resveratrol alone in mice at a 4 mg/kg dosage [77]. 

In another study, resveratrol was encapsulated into a core–shell structure of polymeric nanoparticles formed by mPEG-PCL (poly(ε-caprolactone)–poly (ethylene glycol) and tested in vitro against HT29 and HCT116 colorectal cancer cells. The IC_50_ values of RSV were 47.85 ± 0.96 μg/mL and 23.65 ± 3.21 μg/mL in HT29 and HCT116 cells, respectively (*p* < 0.05). Interestingly, the study indicated that ferroptosis was the cause of cell death induced by resveratrol nanoparticles. Ferroptosis is a form of cell death caused by iron-dependent lipid peroxidation. The study was also extended in vivo to demonstrate the effect on HT 29 xenograft in mice model, where was it demonstrated that after 15 days, the growth of tumors in mice treated with resveratrol formulation co-administered with iRGD (a tumor-penetrating peptide) was much smaller than in the mice treated with saline. The nanoparticles were coated with an erythrocyte membrane, allowing them to escape the macrophage invasion and survive longer in the circulation [78].

D-α-Tocopheryl polyethylene glycol succinate (TPGS) polymer was used to produce polymeric micelles loaded with quercetin. To increase the stability of TPGS polymeric micelles, lecithin was used to form a lipid layer. The stabilized quercetin polymeric micelles were tested in CT26 colon cancer cell lines. The in vitro results showed a lower IC_50_ value, and the in vivo results in Balb/c mice model showed a 360% increased oral bioavailability, as well as reduced tumor size [79]. A pH sensitive polymer Eudragit^®^ S100, loaded with quercetin, was developed by Sunoqrot and Abujamous to target colon cancer cells in vitro. Analysis revealed that H-bonding was responsible for a stable formulation with an IC_50_ of 0.8 μM compared to that of 65.1 μM for free quercetin [80]. In a recent study, quercetin and caffeic acid phenyl ester (CAPE) were coloaded in PLGA nanoparticles and found to increase the apoptotic pathway expression of caspase-3, caspase-9, and other apoptotic genes in HT-29 cell lines. Quercetin nanoparticles not only increased the expression of anti-apoptotic genes such as caspase-3 by 2.38-fold, but they also decreased the IC_50_ values to 11.2 μg/mL after 24 h and 8.2 μg/mL after 48 h [81]. Drug resistance is a problem associated with cancer cells. Shahidi et al. demonstrated that regorafenib resistance in colon cancer cell lines can be reversed by quercetin encapsulated in mPEG-PCL nanoparticles. Regorafenib downregulates the expression of the β1 integrin gene, thereby reducing the tumor cell invasion. Treating the regorafenib-resistant Ls-180 colon cancer cell line with quercetin nanoparticles and regorafenib resulted in the downregulation of the β1 integrin gene by 16.4% in the resistant cells [82].

Chitosan is a thoroughly researched nanocarrier system. Chitosan-based nanoparticles have been exploited as a delivery system for brain-targeted treatment. Chitosan-based nanoparticles can open tight junctions between intestinal epithelial cells and transiently facilitate the paracellular transport of drugs [83]. There are several advantages of using chitosan, i.e., cost, biodegradability, and biocompatibility. Rashedi et al. prepared chitosan-loaded quercetin and 5-FU (fluorouracil) using the ionotropic gelation method and tested this technique on inbred male Wistar rats. The nanoparticles were found to significantly reduce the tumor sizes and increase the apoptotic rate [84].

### 4.3. Gold Nanoparticles

For decades, gold nanoparticles have been an attractive choice for nanoparticle formulations in biotechnology and medical science. Their synthetic chemistry allows for multiple possible interactions through surface functionalization, which can aid in solubility and reduce toxicity. In a novel approach, Kamal et al. designed technetium (Tc)-99m labeled resveratrol-loaded gold nanoparticles and evaluated their targeting efficacy in HT29 colon cancer cells. It was observed that the cancer cell internalization observed for ^99m^Tc-Res-AuNP was significantly higher than that of ^99m^Tc-AuNP and ^99m^Tc-resveratrol. The animal model also showed better in vivo targeting of colon adenocarcinoma with ^99m^Tc-Res-AuNP when compared to ^99m^Tc-resveratrol [85]. A gold nanoparticle core housed in a formulation comprised of quercetin dramatically increased the cytotoxicity by 50-fold in SW-620 colon cancer cells. The in vivo studies showed decreased tumor volume and an altered expression in 27 apoptotic genes [86].

### 4.4. Nanoemulsions

Nanoemulsions are a biphasic dispersion of two immiscible liquids, one in the dispersed phase and the other in the continuous phase. Generally, surfactants and co-surfactants are used as stabilizers and emulsifiers in the two-phase system [87]. Nanoemulsions are usually prepared using high-pressure homogenizers, high-shear stirring, or ultrasound generators [88]. Kotta prepared a nanoemulsion of resveratrol using the phase inversion method and found it to be effective against HCT-116 cancer cell lines at a 30–40 μM concentration [89]. Resveratrol solid lipid nanoparticles, prepared by Serini et al. using a microemulsion technique, were used to encapsulate omega-3 PUFA. The prepared nanoparticle tested against HT-29 cancer cells inhibited the cell growth by 11% compared to its free form. The cell proliferation was significantly inhibited by 50% using the formulation relative to that of the free form; however, this incremental apoptosis could not be co-related to the marginal increase in caspase-3 expression [90]. Similarly, in another study, Feng et al. also demonstrated that the apoptosis efficacy of resveratrol loaded in a lipid core nanocapsule was about 36% in HT 29 cancer cell lines relative to that of free resveratrol [91].

The proinflammatory pathways such as NF-κB are enhanced as a side effect of chemotherapy. Lotfi et al. prepared a nanoemulsion of quercetin using PEG and castor oil and demonstrated that the oxidant–antioxidant balance was achieved in 5-FU induced mucositis in mice. The reversal of destructive histopathology in the colon was achieved because quercetin could decrease the expression of the proinflammatory genes, NF-κB and HIF-1α [92]. In another study, quercetin nanoemulsion, prepared using different oils by high pressure homogenization, was found to have a two-fold lowered IC_50_ of 18 μM for inducing cytotoxicity in HCT-116 and HT-29 colon cancer cell lines. The nanoemulsion did not show any toxic effects on the liver, kidneys, heart, lungs, or brain in mice models at a 50 mg/kg dosage [93].

### 4.5. Dendrimers

Dendrimers are composed of tree-like arms or branches. They are nano-sized and are radially symmetric molecules with a well-defined, homogeneous, and monodisperse structure [94,95,96]. Dendrimers consist of a core with numerous branches arising from the center and are synthesized by repetitive chemical reactions. The two main commercially available dendrimers are Poly (PropyleneImine) PPI and Poly (AMidoAmine) PAMAM [97,98]. Shi et al., using a different approach, tried formulating resveratrol in dendrimers using sugary maize. When tested in a Caco-2 cell line model, it was found to exhibit significantly improved cellular uptake, as well as reduced toxicity. In just half an hour, resveratrol was found to be absorbed, detected on the basolateral side with a Papp value of 9.04 × 10^−6^ cm/s; the intracellular concentration of resveratrol dendrimers was also found to be 1.5-fold more than the free drug level. The resveratrol maize dendrimers were thus found to be absorbable, with an increased bioavailability [99]. A synergistic approach was devised by Ben-Zichri et al., using resveratrol and curcumin in dendrimer formulation. It was found that the noncationic dendrimers loaded with curcumin and resveratrol showed pronounced cancer cell cytotoxicity due to mitochondrial disruption. Intracellular calcium is important for maintaining mitochondrial function and ATP generation. Resveratrol and curcumin dendrimers resulted in a significantly increased intracellular calcium value of 25% in SH-SY5Y cells. This excess calcium disrupted the mitochondrial function, resulting in cell death [100]. Table 2 provides a general summary (brief) of the advantages and limitations of various nanoformulation approaches using phytoconstituents in the treatment of colorectal cancer.

## 5. Synergetic Effect of Co-Loading the Phytoconstituents in Pharmaceutical Formulations

The phytoconstituents focused in this article exhibit similar antioxidant, antiinflammatory, anticancer, and antimicrobial effects, warranting further research into the effect of co-loading these phytoconstituents. As described earlier, the molecular pathways followed by resveratrol and quercetin are diverse. For instance, as shown in Figure 3, resveratrol is a PDE inhibitor, and quercetin is a radical scavenger. Hence, it is imperative to understand the synergetic effect of these phytoconstituents.

The combined effect of quercetin and resveratrol on different types of cancers has been explored in vitro and in vivo. It was demonstrated that quercetin and resveratrol inhibited pancreatic cancer growth, causing apoptosis because of distinct and interacting pathways [101] (Mouria et al.). In another study, it was found that they synergistically induced apoptosis and reduced cell growth in human leukemia cells [102]. Resveratrol and quercetin synergistically triggered apoptosis, inhibiting cell proliferation in glioma cell lines [103]. However, in all these studies, pure phytoconstituents were used, wherein the limitations include solubility and hence, dosage.

Caddeo et al. reported that resveratrol and quercetin liposomes could be used to treat skin inflammation or oxidative stress by significantly reducing edema and leucocyte infiltration [104]. Cadena et al. showed that resveratrol and quercetin elastic liposomes, prepared by incorporating β-cyclodextrin, were shown to exhibit favorable in vitro release kinetics, with an initial burst of drug release of about 50% in the first hour, followed by sustained release of the remaining 50% over 60 h was established. Further studies are required to extend the suitability of such liposomes for reducing subcutaneous fat [105]. Curcumin- and quercetin-loaded nanoemulsions prepared using egg lecithin, castor oil, and fish oil were found to be stable in simulated biological fluids, providing a promising area for further research in its potential against diseases such as cancer. The nanoemulsion was found to be stable, with a negative zeta potential of −25 mV [106]. When co-encapsulated in liposomes, boswellic acids, curcumin, and naringenin reduced the drug dosage capacity by half and exhibited effective in vitro cytotoxicity against Hep G2 liver cell lines [107]. A well-defined ratio of quercetin and vincristine was formulated in liposomes and targeted against estrogen-positive and estrogen-negative breast cancer cells in vitro, showing a significant dose reduction, cytotoxic effect, and sustained release [108]. Zheng et al. showed that the co-loading of curcumin and resveratrol nanoparticles for targeting hepatocellular carcinoma using SP94 mediated delivery could be a promising method to deliver the therapeutic polyphenols for maximum effect. The study showed that curcumin and resveratrol could inhibit the cell growth by more than 40% in vitro, with a promising reduction in tumor volume by more than 30% in vivo compared to the results for free drug molecules [109]. Soltantabar et al. empirically showed that the cytotoxicity of doxorubicin is increased by 20% when coloaded with quercetin in a polymeric formulation. Doxorubicin preferentially accumulated in the nucleus, where it could modulate the DNA, as noted by a DAPI staining technique used in the study [110]. It was shown that a topical gel comprising transferosome, loaded with quercetin and a xanthine oxidase inhibitor, febuxostat, was found to have superior penetration abilities ex vivo and improved success in relieving gout symptoms. The transferosome showed a 33% greater skin penetration than febuxostat gel alone [111].

As elaborated above, quercetin is used in many studies in combination with other drugs, possibly because quercetin is a potent bioenhancer. It could increase the bioavailability of drugs by the inhibition of the Pgp efflux pump and by inhibiting certain metabolic enzymes. For example, in an experiment conducted on chicken ileum, a combination of 100 mg curcumin and 50 mg quercetin was used. The intestinal concentration of curcumin was found to be enhanced by seven-fold, in combination with quercetin in the chicken ileum compared to the result for curcumin alone [112]. Combining the synergistic and therapeutic effects of resveratrol with the bioenhancing capability of quercetin could be a useful strategy.

## 6. Clinical Trials of Resveratrol and Quercetin in Colon Cancer

Based on the information available at ClinicalTrial.gov, a trial (NCT00256334) testing resveratrol as a potential therapeutic for colon cancer treatment was started in 2005. Patients were given commercially available resveratrol tablets at a dose of 80 mg/day, plant derived resveratrol tablets at a dose of 20 mg/day, and grape powder solution in water at a dose of 120 mg/day. The study was performed on patients diagnosed with colon cancer by colonoscopy biopsy and on patients with a plan for surgical resection within 2–4 weeks of enrollment. However, to date, the results of this study have not yet been published [113].

There are multiple ongoing clinical trials to investigate the anti-cancer properties of quercetin [114]. However, most of them, although completed, do not yet show available results. For colon cancer, an optimal dose of QUE (one of three doses orally, twice a day, for 6 to 10 weeks) that is effective in modulating the biomarkers of colon epithelial cell turnover and therefore, potentially inhibiting colon cancer development, was studied. However, although this trial (NCT00003365) was completed in 2006, no results have been revealed [115]. Grape juice containing resveratrol and QUE was evaluated, among other dietary interventions (NCT00455416). The study completion date was 2009, but no results have yet been published [116].

## 7. Toxicity and Production Feasibility

The kidney and the liver are the two major organs highlighted in toxicity profiling for pharmaceuticals. Urea and creatinine are markers for renal failure, while ALT (alanine transaminase) and AST (aspartate aminotransferase) levels provide a first-hand picture of hepatoxicity caused by any drug or formulation. A study evaluated the potential of resveratrol liposomes in treating breast cancer, along with their toxicity. It was found that the encapsulated resveratrol did not show any physical impairment in the treated animals compared to the control group. The urea, creatinine, ALT, and AST values in the treated group were found to be like those of the control group, indicating no renal or hapatic toxicity in the animals treated with resveratrol liposomes. In fact, unformulated resveratrol showed elevated levels of creatinine at 80 µmol/L and enzyme ALT at 120 IU/L compared to resveratrol liposomes at 60 µmol/L and 80 IU/L, respectively [70] This clearly indicates that the phytochemical that could potentially be toxic is safe when encapsulated in a nanoformulation. In another study, it was demonstrated that resveratrol encapsulated in folate-conjugated human serum albumin (HSA) showed a marginal increment in the weight, without any organ toxicity [117]. Samir et al. demonstrated the non-toxic nature of resveratrol invasome gels formulated to treat skin cancers in vivo. The levels of urea and creatinine in the treated groups were 40 and 0.7 ng/mL relative to 55 and 0.7 ng/mL in the control group. Similarly, the levels of ALT and AST were reported to be 153 and 289 ng/mL compared to 151 and 410 ng/mL, respectively, in the control group [118]. Quercetin niosomes were found to significantly reduce the carbon tetrachloride-induced hepatotoxicity in animals. The liver biomarker enzymes ALT and AST were reduced by 61% and 49%, respectively, upon treatment with quercetin niosomes for five days [119]. Quercetin nanoformulation in PLGA was found to have a therapeutic effect on mammary adenocarcinoma in rats. The formulation had no toxic effect on the animals, as shown by body weight, which was found to be 278 g compared to 312 g in the control groups [120]. These studies suggest that nanoformulations of resveratrol and quercetin exhibit non- toxic characteristics and thus warrant further exploration in human trials.

The appropriate composition of the formulation, the functional excipients, preparation techniques, and process parameters determine the production feasibility. While creating the novel drug delivery formulation, researchers should concentrate on and optimize investigations that evaluate the product’s size, appearance, stability, and drug release. Additional analytical data allow for an evaluation of the production process and formulation methods to optimize yield, lower expenses, and guarantee the quality and reproducibility of the final product [121]. Computational models can be used to justify the viability of utilizing innovative formulations to improve medication administration and lessen the need for preclinical and clinical research. In the early phases of drug discovery and development, the use of model-informed drug discovery and development (MID3) techniques can assist in defining ideal drug characteristics for a target product profile, prioritizing targets, and assessing the viability of drugging a target. Early feasibility assessment (EFA) provides a great deal of potential to realize efficiencies and lower attrition in drug development when applied in a formulation method before the significant clinical costs are incurred [122]. Although these phytoconstituents have a reputation for being healthful, the cost of the production, extraction, and purification methods can affect the finished product’s price. Furthermore, consumers looking to optimize health advantages may find cost-effective solutions in the possible synergistic effects of mixing quercetin with resveratrol. By targeting the administration of the resveratrol and quercetin combination to the tissues, this novel delivery system maximizes their therapeutic effect. The protective qualities of novel formulation may also result in improved product stability, potentially extending shelf life and reducing wastage, further enhancing the economic feasibility of this novel delivery system for the pharmaceutical company.

## 8. Conclusions and Future Perspective

Fundamentally, the available literature provided evidence that resveratrol and quercetin are potent for tackling various pathologies in colon cancer using various mechanistic pathways. The therapeutic potential of resveratrol and quercetin is also well established by their abilities to reduce tumor growth, inhibit metastasis, and increase the apoptotic, antiinflammatory, and antioxidant pathways. The bioavailability of lipophilic resveratrol and quercetin is effectively tamed by their encapsulation in different formulations, such as liposomes, nanoemulsions, polymeric micelles, nanoparticles, and dendrimers. Recent studies have also shown promising results in improving the bioavailability of resveratrol and quercetin nanoformulations in in vivo studies of colon cancer. This area of research is very dynamic, with vigorous advancements in terms of designing synthetic nanoparticles and creating more targeted nanoformulations for colon cancer treatment. Quercetin and other polyphenols are already involved in the preclinical stage of development for the treatment of CRC. Resveratrol, on the other hand, is included in a phase I clinical trial as a candidate for the treatment of colon cancer. A combination of resveratrol and quercetin has shown synergistic effects in combating inflammation. In the future, quercetin, which is an established bioenhancer, could be effectively used in combination with resveratrol as a potential therapeutic drug with enhanced bioavailability for combating colorectal cancer.

## Figures and Tables

**Table 1:** Common chemical properties of resveratrol and quercetin that contribute towards the pharmacological mechanisms targeting colon cancer.

**Description**	**Resveratrol**		**Quercetin**	
**Chemical Properties** [57,58]				
Solubility	PEG-400 (373 mg/mL), Ethanol (88 mg/mL), Labrasol (14 mg/mL), Tween 80 (7 mg/mL), Cremophore EL (6.7 mg/mL), Polysorbate 80 (5 mg/mL), water (0.05 mg/mL)		DMSO (67 mg/mL), Ethanol (21 mg/mL), Acetone (80 mmol/L), t-amyl alcohol (67 mmol/L), Acetonitrile (5.4 mmol/L), water (0.4 mg/L)	
Stability	Unstable at high temperatures and at alkaline pH		Unstable at higher temperatures and at alkaline pH	
**Pharmacological Targets in CRC** [23,24,25,26,27,28,29,30,31,32,33,34,35,51,52,53,54,55,56]				
**Pathways/Molecules**	**Upregulated**	**Downregulated**	**Upregulated**	**Downregulated**
Anti-cancer	SIRT1, p38-MAPK, PDH,	MMP-9, VEGF, EMT, HIF-1alpha, TSP1, NO, MMP-2, iNOS, AKT/mTOR, Wnt,	P53, BAX, caspase-3, and caspase-9,	Wnt/β-catenin, PI3K/AKT, p-STAT3, NF-κB,

**Table 2:** A general summary (brief) of the advantages and limitations of various nanoformulation approaches for phytoconstituents in the treatment of colorectal cancer.

Description	Liposomes	Nanoemulsions	Dendrimers	Polymeric Micelles
Advantages	Lipids are used in the formulation Slow drug release Enhanced drug stability Surface modifications possible High drug encapsulation efficiency Biodegrdable and biocompatible	Two immiscible liquids are used in preparation Larger surface area for enhanced absorption Enhanced drug stability Lotion, spray, liquid, and foam formulation possible Biodegrdable and biocompatible	Structural versatility Enhanced drug solubility and bioavailability Enhanced drug stability Surface modifications possible High drug encapsulation efficiency Biodegrdable and biocompatible	Amphiphilic copolymers used in preparation Low cost Enhanced drug stability High drug encapsulation efficiency Site-specific drug delivery; high cellular uptake Tailored drug release pattern Good pharmacokinetic behavior
Limitations	Rapid clearance Aggregation Drug leakage Low cellular uptake, depending on the formulation protocols Lipids are used in the formulation, and interindividual variation occurs	Temperature and pH can impact stability Unsuitable for drugs with high melting points Robust surfactant concentrations are required Surfactant toxicity Phase separation in long-term storage Cost of production is high	Interaction with cell membrane leading to cell death Neurotoxic, renal, hepatic, and gastrointestinal toxicity	Burst release Surface changes not possible
